# Pooled Oncological Outcomes in Marjolin’s Ulcer: A Systematic Review and Meta-Analysis

**DOI:** 10.7759/cureus.113191

**Published:** 2026-07-22

**Authors:** Saikat Das, Jerene Mathews, Aswathy Anilkumar, Anvita G Malhotra, Gaurav Chaturvedi, Ajitesh Avinash, Suparna K Pal, Atreyee Ghosh, Amit Agrawal

**Affiliations:** 1 Department of Radiation Oncology, All India Institute of Medical Sciences Bhopal, Bhopal, IND; 2 Department of Dermatology, All India Institute of Medical Sciences Bhopal, Bhopal, IND; 3 Center for Health and Technology Assessment, All India Institute of Medical Sciences Bhopal, Bhopal, IND; 4 Department of Burns and Plastic Surgery, All India Institute of Medical Sciences Bhopal, Bhopal, IND; 5 Department of Radiation Oncology, SUM Ultimate Medicare, Siksha 'O' Anusandhan Deemed to be University, Bhubaneswar, IND; 6 Department of Radiation Oncology, Institute of Post Graduate Medical Education & Research, Kolkata, IND; 7 Indian Institute of Technology Indore (IITI) Driving Innovation Through Simulation Hub for Technologies in Interdisciplinary Cyber-Physical Systems (DRISHTI CPS) Foundation, Indian Institute of Technology Indore, Indore, IND; 8 Department of Neurosurgery, All India Institute of Medical Sciences Bhopal, Bhopal, IND

**Keywords:** burn wounds, marjolin’s ulcer, post burn neoplasm, skin cancer, wound malignancy

## Abstract

Marjolin's ulcer is a rare, aggressive cutaneous malignancy arising in chronic wounds, scars, and burns, for which prognostic and treatment data remain fragmented across small retrospective series. We conducted a systematic review and meta-analysis, registered with the International Prospective Register of Systematic Reviews (PROSPERO) and performed according to Preferred Reporting Items for Systematic Reviews and Meta-Analyses (PRISMA), searching PubMed, EMBASE, Scopus, the Cochrane Library, and ScienceDirect for case series and cohort studies (≥10 patients) reporting histologically confirmed malignancy arising in scars, ulcers, or chronic wounds. Pooled proportions were estimated using random-effects models (restricted maximum likelihood (REML)) with logit transformation, treatment arms were compared using log risk ratios, and risk of bias was assessed with the Joanna Briggs Institute (JBI) checklist. Fifty-one studies comprising 2,388 patients across 18 countries were included. Squamous cell carcinoma predominated (95.7%), arising chiefly in the lower limb (58.9%) after a long latency (median 29.5 years). The pooled local recurrence rate was 20% (95% confidence interval (CI) 16%-24%; I² = 66.3%), while the pooled rate of regional nodal metastasis at diagnosis was 19% (95% CI 15%-24%; I² = 78.5%); the pooled five-year overall survival was 61% (95% CI 49%-72%). Adding adjuvant radiotherapy to surgery showed no significant effect on local recurrence (log risk ratio 0.78, 95% CI −1.56-3.12), though confounded by indication. Evidence certainty was low to very low; wide local excision with clear margins, according to the oncological principles, remains the cornerstone of management.

## Introduction and background

Historically, Marjolin's ulcer (MU) referred to malignant transformation occurring within an old burn scar; the term is now used more broadly to encompass any malignant tumor arising in a chronic cutaneous ulcer, area of chronic inflammation, or burn scar [1]. MU was first described by Jean-Nicolas Marjolin in 1828 as a "verrucous ulcer" without identifying its malignant nature [2-4]. In 1850, Robert Smith linked the development of carcinoma in scars to Marjolin's warty ulcers. The eponym "Marjolin's ulcer" was subsequently introduced by Da Costa in 1903 [5] to describe the malignant transformation of chronic non-healing ulcers that have developed in burn scars or in other chronic wounds [6]. About 2% to 6% of burn scars transform to MU [7]. Squamous cell carcinoma (SCC) remains the most common histology, but other cutaneous cancer types, including basal cell carcinoma (BCC), melanoma, and sarcoma, have also been identified in the inflamed or injured tissue, caused by burn, scald, trauma, osteomyelitis, chronic ulcer, or wounds [8]. Approximately 2% of SCC and 0.03% of BCC arise from post-burn MU [9]. Criteria for defining scar carcinoma have been proposed by Segond et al. [10]. Though rare, MU is caused by pathological changes resulting from prolonged repetitive trauma after full-thickness skin loss, due to poor wound healing, repeated injury, improper wound care leading to malignant transformation of burns, chronic non-healing wounds, long-term fissures, ulcers, lupus vulgaris, vaccine scars, osteomyelitis zones, and post-radiotherapy (post-RT) areas [11]. MU is a virulent form of skin cancer compared to non-Marjolin’s SCC [12]. Because of its rarity, a clearly defined treatment protocol is lacking. Features such as primary tumor size, presence of metastatic lesions from the primary tumor, depth of invasion, and degree of differentiation appear to be prognostically important. Surgical treatment remains the mainstay of treatment; the role of prophylactic lymph node dissection, sentinel node biopsy, and adjuvant treatment, including RT, is not well defined in the literature. To our knowledge, no consensus exists on the planning and sequencing of multidisciplinary management for this condition [13]. While several reviews on MU have been published in the literature [1,14-21], our study advances this evidence base by providing a focused, quantitative synthesis of the patient-, tumor-, and treatment-related factors that influence oncological outcomes. By pooling the available studies, we provide more precise, evidence-based estimates to inform prognostication and management of this aggressive malignancy. Although prior systematic reviews have addressed MU [22], considerable variability in its characteristics, prognosis, and treatment options precludes consensus, and there is a lack of large-scale synthesis evidence on MU addressing oncological outcomes [1,14,18,23].

## Review

Materials and methods

This systematic review was conducted following the Preferred Reporting Items for Systematic Reviews and Meta-Analyses (PRISMA) guidelines [24] and the Cochrane Manual of Systematic Reviews and Meta-analyses [25]. The protocol was registered in the International Prospective Register of Systematic Reviews (PROSPERO) database (PROSPERO 2026 CRD420261424320).

Search Strategy and Information Sources

We searched PubMed, EMBASE, Scopus, the Central Cochrane Registry of Controlled Trials (The Cochrane Library), and the ScienceDirect database with search terms (Appendix). In addition, the reference lists of included studies were evaluated for potentially eligible studies; the full texts of these articles were obtained online or via communication with the authors.

Inclusion Criteria

All identified references were imported into EndNote™ reference manager software (Clarivate, London, UK). After removing duplicates, two independent reviewers screened the titles and abstracts for eligibility based on predefined criteria. Case series and cohort studies (retrospective and prospective) describing the clinicopathological presentation and management of MU were included (provided they included ≥10 patients). Studies describing histopathologically confirmed malignancies arising from chronic scars, ulcers, or sinuses with variable lag periods were included. Eligible studies included case series and observational studies reporting histologically confirmed skin cancer arising from burn scars of any etiology (flame, scald, electrical, friction, or infection-related). Eligible studies included primary research, case series (≥10 cases), and observational studies that reported any type of skin cancer occurring subsequent to various burn scars (flame, trauma/surgery, stasis ulcer, scald, infection, and electric burn).

Exclusion Criteria

We excluded studies involving non-human subjects, non-cancerous skin injuries without histologically confirmed malignancy at the time of initial injury, secondary studies, or those that could not be translated into English. Pseudo-epitheliomatous hyperplasia, atypical proliferation with no histopathologically proven malignancy, case series with <10 patients, and review articles were excluded.

Data Extraction and Outcome Analysis

Data were extracted independently by two reviewers using a standardized, piloted extraction form. Discrepancies were resolved by discussion and consensus; where agreement could not be reached, a senior author adjudicated the final decision. For each included study, the following variables were extracted. Study characteristics comprised the first author, year of publication, country, and study design. Patient characteristics included sample size, age, and sex. Wound- and burn-related data comprised the etiology of the antecedent wound (flame, scald, electrical or chemical burn, trauma or surgery, friction, or chronic infection), the anatomical site of injury (head and neck, face, trunk, upper limb, lower limb, or other), and the latency period. Tumor-related data comprised the histological type of malignancy (SCC, BCC, or others), locoregional spread, the treatment modality (wide local excision (WLE), amputation, lymph node dissection, RT, or chemotherapy), and the quality-of-life (QoL) score recorded (if any). The prespecified outcomes were the duration of follow-up and oncological outcomes of local recurrence, regional lymph node metastasis, distant metastasis, and five-year survival.

For the quantitative synthesis, the primary outcomes were the pooled proportions of local recurrence, regional nodal metastasis, distant metastasis, and five-year overall survival. Secondary analyses examined the role of adding RT to surgical treatment. Effect estimates (risk ratios with 95% confidence intervals (CIs)) and the underlying contingency-table data were extracted whenever available to permit quantitative pooling; where only descriptive statistics or p-values were reported, the direction and statistical significance of the association were recorded for narrative synthesis. Quantitative extraction was carried out in Microsoft Excel (Version 16, Microsoft Corp., Redmond, WA, USA), and all quantitative data synthesis and meta-analyses were performed using the R statistical computing environment (Version 4.6.1, R Core Team, Vienna, Austria) via RStudio. Pooling was performed in the meta package using the random-effects model (restricted maximum likelihood (REML)). Logit-transformed proportions were used, and intergroup differences were assessed using log risk ratios. Heterogeneity levels were assessed using I^2^, tau^2^, and the Chi-squared test.

Risk of Bias Assessment

Risk of bias was assessed using the Joanna Briggs Institute (JBI) Critical Appraisal Checklist for Case Series [26].

Results

Study Screening

**Figure 1:**
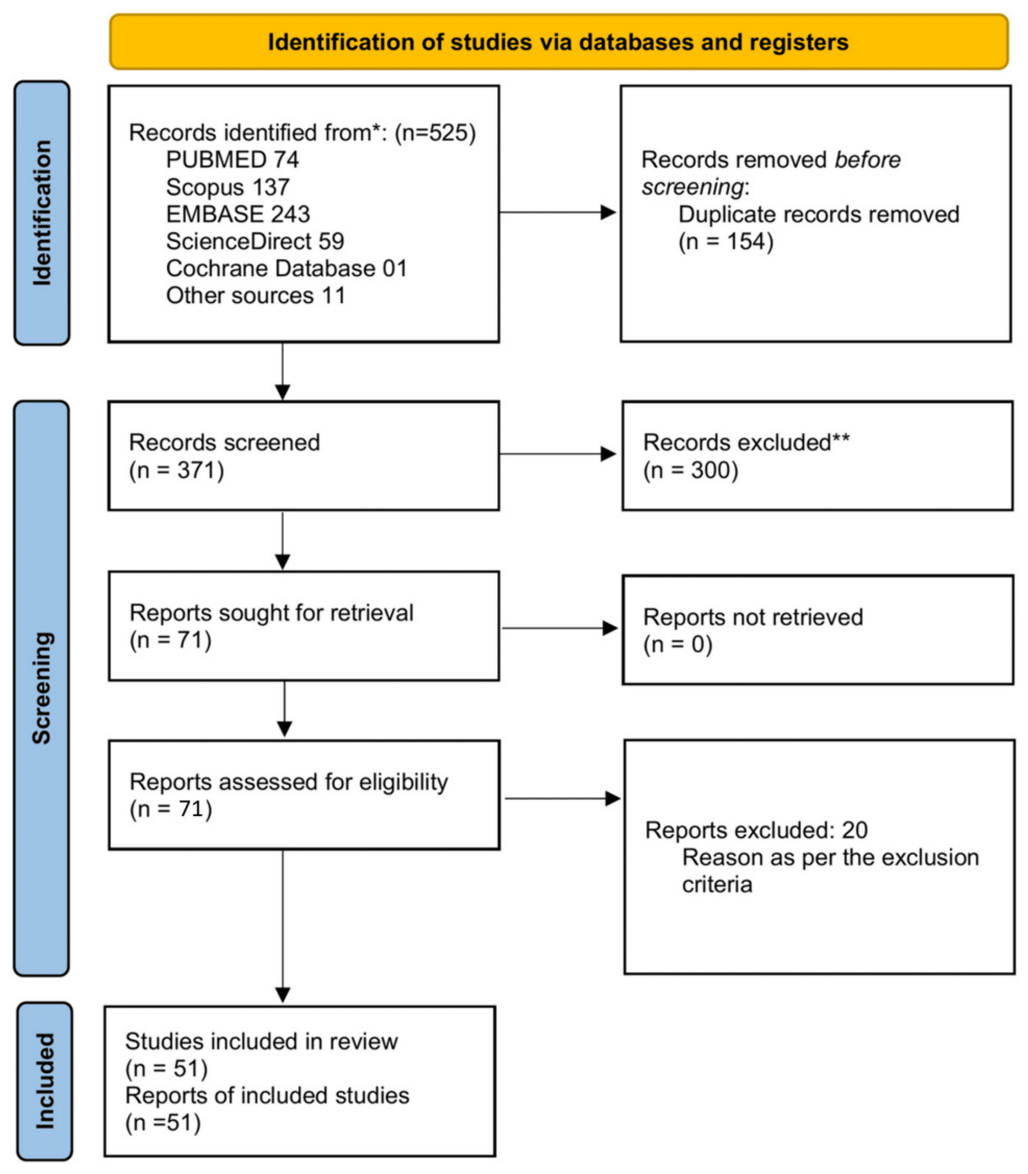
PRISMA 2020 flow diagram for the systematic review, which included searches of databases, registers, and other sources Source of the template: Page et al. [24] (Creative Commons Attribution 4.0 International (CC BY 4.0)) PRISMA: Preferred Reporting Items for Systematic Reviews and Meta-Analyses

A total of 525 records were retrieved from the searched databases and were screened by two independent reviewers based on titles and abstracts. Of these, 371 records were deemed eligible for full-text screening after removing 154 duplicates. Three hundred unrelated studies were excluded; 71 studies underwent full-text screening, resulting in the inclusion of 51 studies in the current systematic review [6,7,9-13,15,23,27-68]. The PRISMA flow diagram representing the study selection process and number of records is shown in Figure 1.

Patient-, Tumor-, and Treatment-Related Characteristics

**Figure 2:**
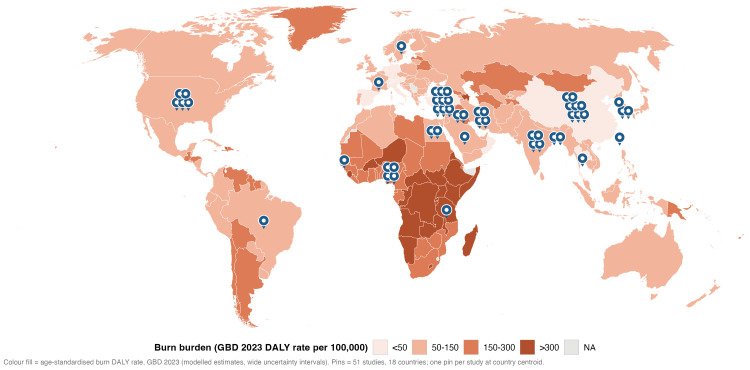
Global burn disease burden versus country-wise distribution of included studies The color fill represents the age-standardized disability-adjusted life year (DALY) rates for burns (fire, heat, and hot substances) per 100,000 population, sourced from the Global Burden of Disease Study 2023 (GBD 2023) [69]. Each pin denotes the country of origin for one of the included studies (51 studies across 18 countries). The included studies are concentrated in low- to moderate-burden countries, with a predominance of English-language publications, while the highest-burden countries in central and eastern Africa are underrepresented or absent. Figure generated by the authors using RStudio, Version 2026.05.0+218 (Posit Software, Boston, MA, USA).

**Figure 3:**
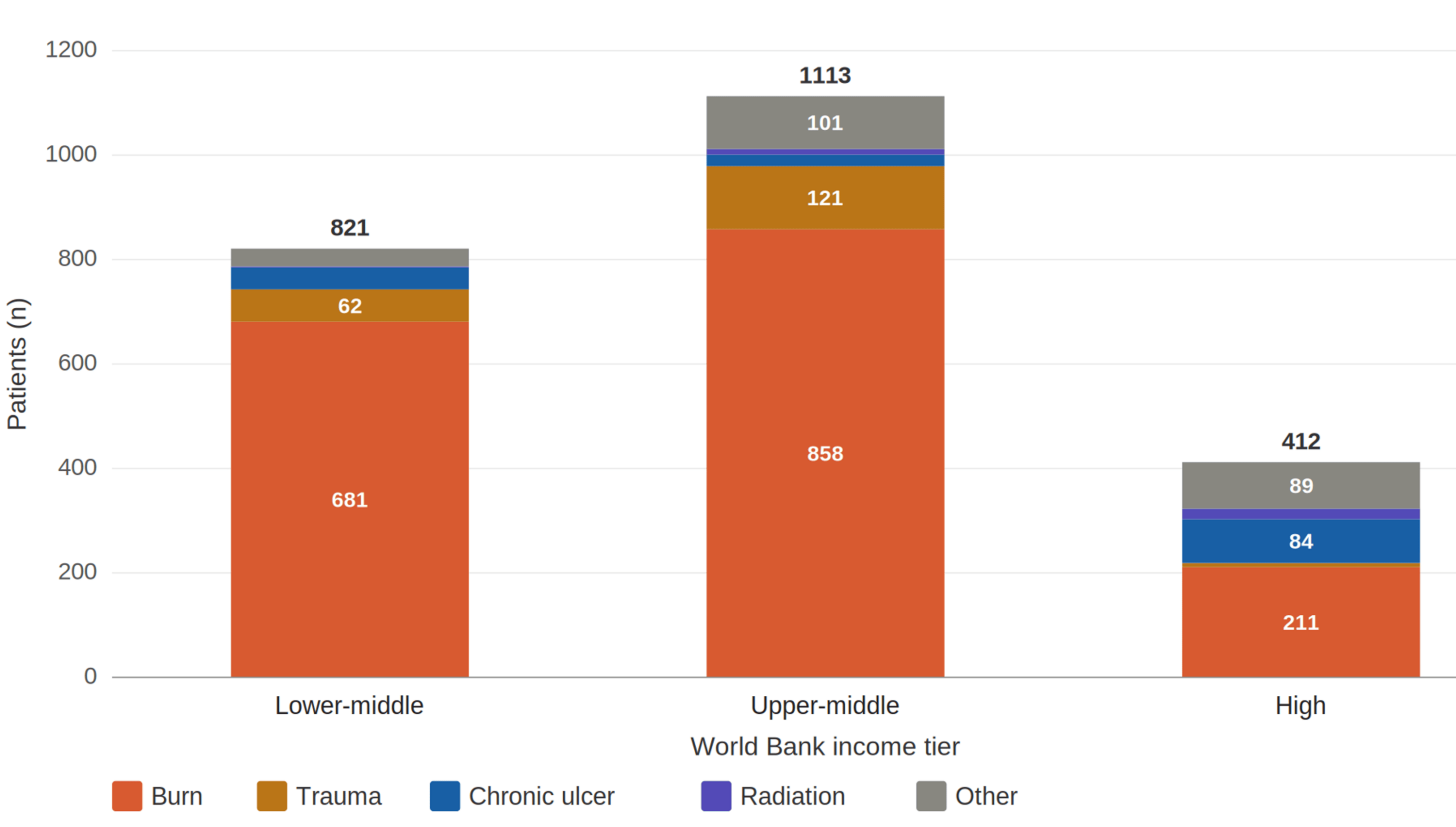
Etiology of Marjolin's ulcer by World Bank income tier (absolute patient counts; 51 studies, 18 countries, 2,346 patients) Burn predominates overall and in the lower- and upper-middle tiers, while other causes-notably chronic venous ulcers-contribute proportionally more in high-income countries. No low-income country contributed a study.

A total of 51 studies published between 1965 and 2025 were included, comprising 2,388 patients across 18 countries (Table 1, Figures 2, 3).

**Table 1:** Characteristics of the included studies SCC: squamous cell carcinoma; BCC: basal cell carcinoma

#	Study	Year	Country	Total number of patients in the study	Mean age (y)	Etiology, n					Predominant site, n				Latency (y)	Histology, n			
						Burn	Trauma	Ulcer	Radiation	Other	Lower	Head/neck	Upper	Trunk		SCC	BCC	Melanoma	Other
1	Al-Zacko [27]	2013	Iraq	27	42.7	27	0	0	0	0	22	2	3	0	27.6	27	0	0	0
2	Arons [10]	1965	USA	22	58.5	22	0	0	0	0	9	7	4	2	36.5	22	0	0	0
3	Aydoğdu [28]	2005	Turkey	15	51.05	15	0	0	0	0	5	5	0	0	40.9	15	0	0	0
4	Kumar [49]	2024	India	27	52	17	9	0	0	1	16	5	5	1	11	23	3	0	1
5	Baldursson [29]	1999	Sweden	25	78.5	25	0	0	0	0	25	0	0	0	>3	25	0	0	0
6	Bang [6]	2018	South Korea	14	47.5	12	0	2	0	0	14	0	0	0	NR	14	0	0	0
7	Barr [12]	1983	USA	37	64	18	0	12	0	7	28	0	7	2	36	37	0	0	0
8	Bozkurt [11]	2010	Turkey	16	57.1	16	0	0	0	0	9	2	2	3	42.3	14	2	0	0
9	Burusapat [23]	2021	Thailand	14	59.7	11	1	0	0	2	10	0	1	3	27.8	13	0	0	1
10	Challa [13]	2014	India	14	50	9	1	1	0	3	9	1	2	2	4.85	14	0	0	0
11	Chalya [30]	2012	Tanzania	56	38.2	50	0	1	0	5	26	13	12	4	11.34	51	2	1	2
12	Chaturvedi [31]	2019	India	55	48.75	42	10	0	0	3	38	5	6	6	26.4	55	0	0	0
13	Chen [32]	2020	China	42	53.6	30	12	0	0	0	0	42	0	0	39.7	42	0	0	0
14	Combemale [33]	2007	France	80	75	0	0	70	0	0	80	0	0	0	NR	83	2	0	0
15	Copcu [34]	2003	Turkey	31	39	30	0	0	0	1	18	6	0	6	19	16	4	0	1
16	Das [35]	2015	Bangladesh	46	45.15	45	0	0	0	1	18	4	14	10	26.73	46	0	0	0
17	Edwards [36]	1989	USA	66	58	46	0	0	20	0	NR	NR	NR	NR	37	66	0	0	0
18	Ehsani [9]	2016	Iran	30	59.2	27	2	0	0	1	16	4	8	2	32.4	30	0	0	0
19	Eroğlu [37]	1997	Turkey	91	51	66	0	0	11	14	42	23	13	13	32	91	0	0	0
20	Fahim [38]	2022	Bangladesh	30	40.47	26	0	0	0	4	19	1	10	0	19.5	19	0	0	0
21	Ghalambor [39]	2007	Iran	148	46	148	0	0	0	0	131	0	16	0	6-12	148	0	0	0
22	Gül [7]	2006	Turkey	36	46.8	36	0	0	0	0	20	8	4	4	32	36	0	0	0
23	Guo [40]	2018	China	40	53	29	7	0	0	4	23	15	0	0	NR	35	2	0	2
24	Gupta [41]	2023	India	66	40	66	0	0	0	0	66	0	0	0	NR	66	0	0	0
25	Hahn [42]	1990	South Korea	19	50.2	10	2	0	0	7	16	2	0	1	31.5	19	0	0	0
26	Huang [43]	2010	Taiwan	11	47	11	0	0	0	0	6	1	4	0	27.3	10	0	0	1
27	Jiburum [44]	2016	Nigeria	70	44.9	14	16	40	0	0	50	0	0	0	22.1	66	0	2	0
28	Kadir [45]	2007	Iraq	48	40	48	0	0	0	0	14	16	0	0	30	48	0	0	0
29	Kasse [47]	1999	Senegal	67	41	67	0	0	0	0	59	NR	NR	6	27	65	0	0	2
30	Kerr-Valentic [48]	2009	USA	10	57.5	4	3	0	0	3	0	2	4	4	34.6	10	0	0	0
31	Liu [51]	2016	China	187	49	133	28	0	0	24	82	80	15	10	32	187	0	0	0
32	Lifeso [50]	1990	Saudi Arabia	63	51	16	0	0	0	47	56	0	7	0	17.8	63	0	0	0
33	Luo [52]	2025	China	126	51.5	100	12	0	0	0	60	42	17	4	34.4	116	6	0	0
34	Metwally [53]	2017	Egypt	26	47	24	2	0	0	0	20	0	5	1	25	26	0	0	0
35	Mousa [54]	2022	Egypt	19	55.4	19	0	0	0	0	12	4	3	2	29.68	19	0	0	0
36	Onah [55]	2006	Nigeria	29	42	6	6	0	0	4	NR	NR	NR	NR	12	29	0	0	0
37	Oruç [56]	2017	Turkey	63	49.7	52	8	0	0	3	33	16	7	7	37.9	63	0	0	0
38	Ozek [57]	2001	Turkey	40	46.5	40	0	0	0	0	23	5	5	7	34.3	40	0	0	0
39	Ozinko [58]	2017	Nigeria	13	13	3	10	0	0	0	9	1	2	1	38	9	1	0	1
40	Sadegh Fazeli [59]	2013	Iran	83	55.3	74	0	0	2	7	55	18	10	0	NR	68	0	2	5
41	Shahla [60]	2009	Iran	19	29.5	19	0	0	0	0	19	0	0	0	11	19	0	0	0
42	Shen [61]	2015	China	51	64.2	35	16	0	0	0	31	4	7	1	13.42	43	1	6	1
43	Smith [62]	2001	Brazil	21	76	0	0	21	0	0	21	0	0	0	NR	21	0	0	0
44	Stromberg [63]	1977	USA	31	58	3	3	0	0	25	21	0	0	0	23	31	0	0	0
45	Tiftikcioglu [64]	2010	Turkey	62	48	62	0	0	0	0	48	0	14	0	35.9	62	0	0	0
46	Tahir [15]	2012	Nigeria	36	40.8	25	6	NR	NR	5	19	5	7	5	22.9	34		1	1
47	Xiao [66]	2019	China	31	52.2	19	7	0	0	5	0	31	0	0	42.9	27	0	0	4
48	Xiang [65]	2019	China	140	116	78	20	0	0	42	61	50	13	10	28.8	123	10	0	7
49	Karasoy Yesilada [46]	2013	Turkey	34	50	23	5	0	0	6	15	6	4	9	38.5	31	0	1	2
50	Yoo [67]	2014	South Korea	44	52.5	44	0	0	0	0	30	2	7	5	32	42	0	0	2
51	Yu [68]	2013	China	17	62	8	5	1	0	0	4	4	0	0	29	17	0	0	0

The great majority were single-institution retrospective case series, and individual study size varied widely, from fewer than 15 patients to series of 126-187 cases. The reported mean age generally fell within the fifth to seventh decades of life. Burns were the predominant antecedent injury, accounting for roughly three-quarters of all cases in which etiology was specified (1,725 of 2,310 coded cases, 74.7%), with trauma, chronic ulcers, and post-radiation scars making up most of the remainder. The lower limb was the most commonly affected site (1,389 of 2,173 coded cases, 63.9%), followed by the head and neck, upper limb, and trunk. SCC was the dominant histological subtype, representing well over 90% of tumors, while BCC, melanoma, and other rare malignancies (including sarcomas and adnexal tumors) were reported only sporadically. The interval between the original injury and malignant transformation was long in most series, typically spanning two to four decades.

**Figure 4:**
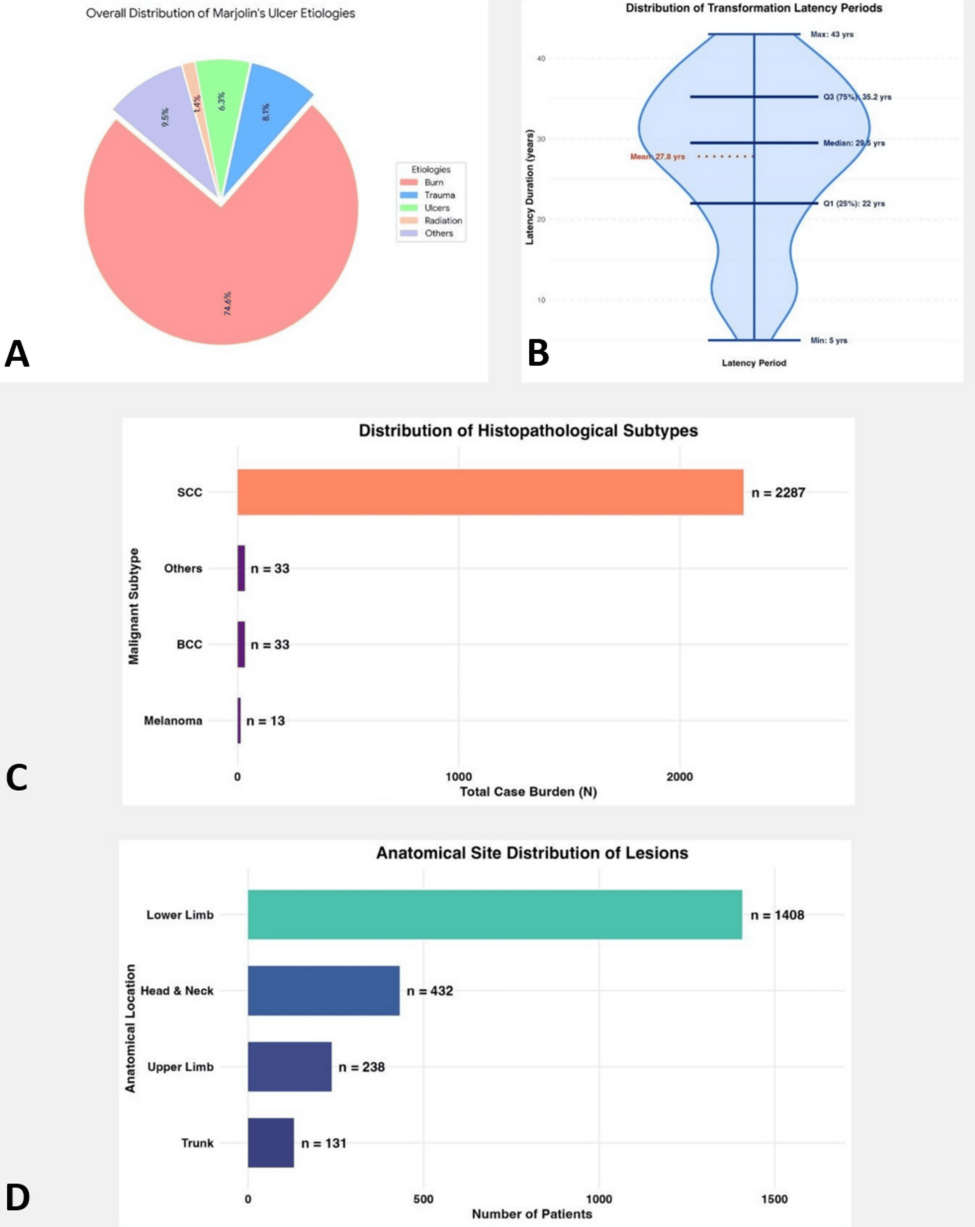
(A-D) Distribution of etiology, latency period, histology, and site

The aggregate clinicopathological profile of the included cohort is summarized in Figure 4. Burn was the most common etiology (Figure 4A). The latency between the inciting injury and malignant transformation was characteristically long and widely dispersed, with a median of 29.5 years (Figure 4B). Histopathologically, SCC was overwhelmingly predominant, comprising 2,287 tumors, with BCC (n = 33, 1.4%), other rare malignancies (n = 32, 1.4%), and melanoma (n = 13, 0.5%) together representing fewer than 4% of cases (Figure 4C). The anatomical distribution showed a clear predilection for the lower limb, accounting for 1,408 cases, far exceeding those of the head and neck (n = 432), upper limb (n = 238), and trunk (Figure 4D).

Evidence Synthesis and Meta-Analysis

**Figure 5:**
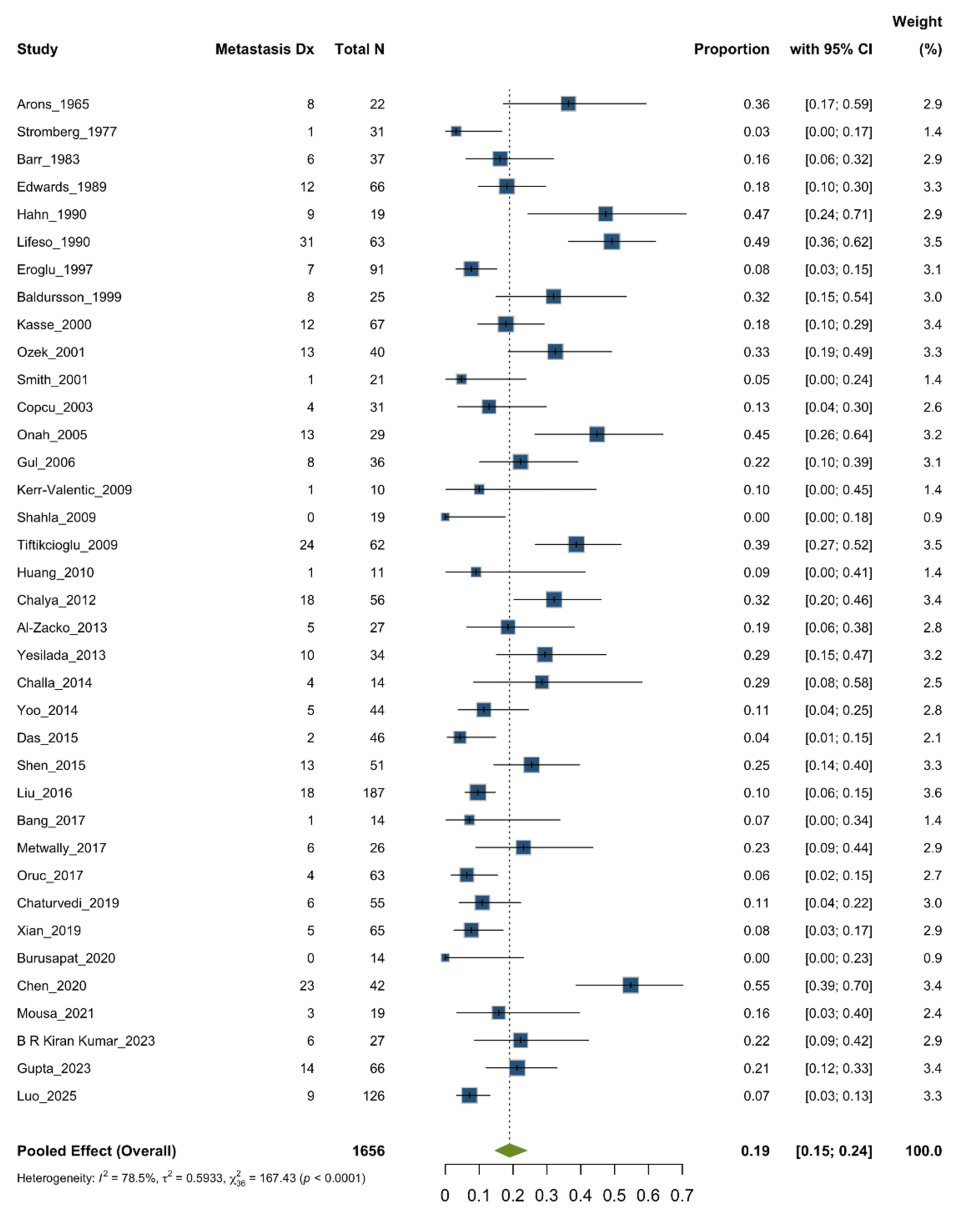
Forest plot of lymph node metastasis at diagnosis across studies References [6,7,10,12,13,23,27,29-32,34-37,41-43,46-51,53-57,60-65,67] CI: confidence interval

Nodal metastasis at the time of diagnosis was reported in 37 studies (1,656 patients), with a pooled prevalence of 19% (95% CI 15%-24%); between-study heterogeneity was substantial (I² = 78.5%, τ² = 0.59, χ²₃₆ = 167.43, p < 0.0001, Figure 5).

**Figure 6:**
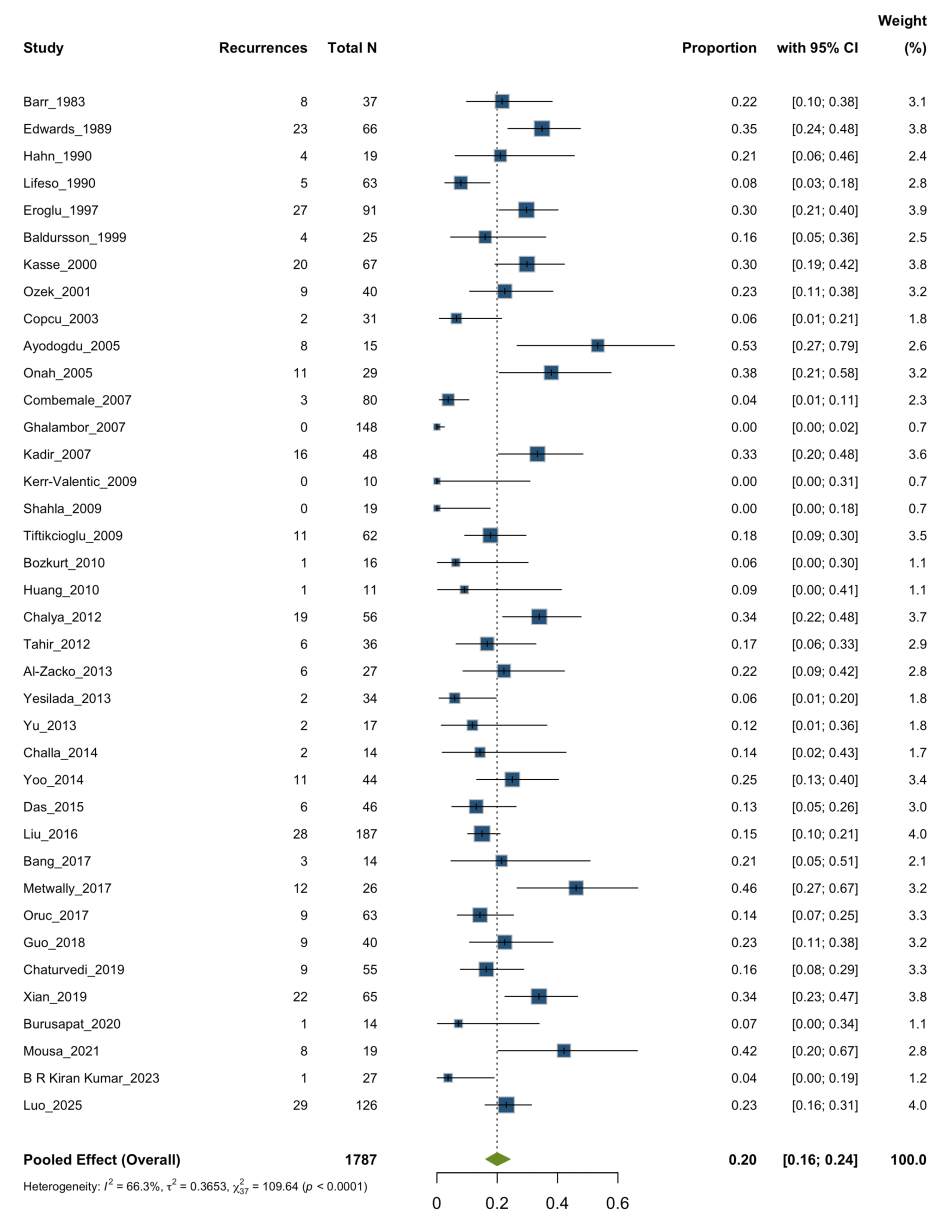
Forest plot of local recurrence across studies References [6,11-13,15,23,27-31,33-37,39,40,42,43,45-57,60,64,65,67,68] CI: confidence interval

A total of 2,181 patients underwent surgery (92.7%). RT in any setting (post-op/only RT/palliative) was used in 9.3% of patients. Local recurrence (Figure 6) was assessed across 38 studies (1,787 patients) and showed a comparable pooled proportion of 20% (95% CI 16%-24%), with moderate-to-substantial heterogeneity (I² = 66.9%, τ² = 0.38, χ²₃₆ = 108.81, p < 0.0001).

**Figure 7:**
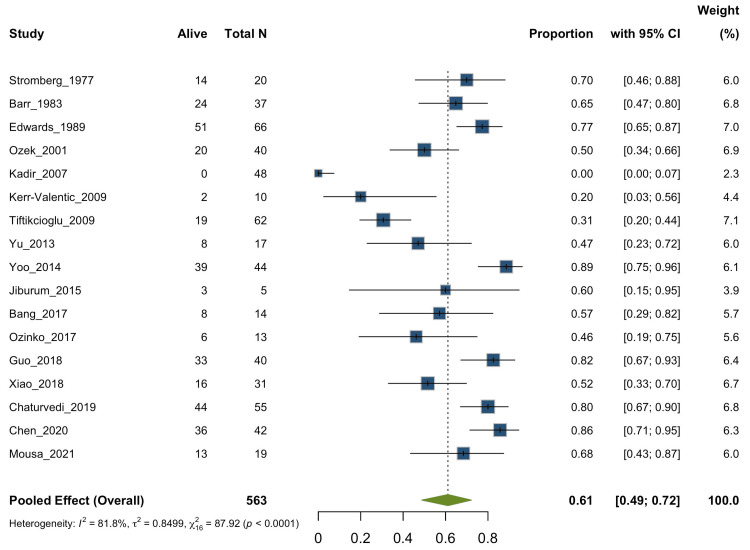
Forest plot of five-year survival across studies References [6,12,31,32,36,40,44,45,48,54,57,58,63,64,66-68] CI: confidence interval

Pooled five-year overall survival, derived from 17 studies (563 patients), was 61% (95% CI 49%-72%), again with considerable heterogeneity (I² = 81.8%, τ² = 0.85, χ²₁₆ = 87.92, p < 0.0001, Figure 7).

**Figure 8:**
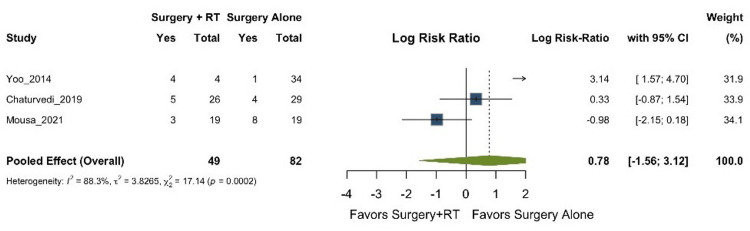
Comparison of surgery vs. surgery + radiotherapy (RT), based on available information in the studies References [31,54,67] CI: confidence interval

Comparison of surgery alone versus surgery plus adjuvant RT was possible in only three studies (49 patients treated with surgery + RT versus 82 treated with surgery alone, Figure 8) and yielded a pooled log risk ratio of 0.78 (95% CI −1.56 to 3.12); the CI crossed the line of no effect, indicating no statistically significant difference between the two strategies, with very high heterogeneity across the contributing studies (I² = 88.3%, τ² = 3.83, χ²₂ = 17.14, p = 0.0002).

No published research study was available that reported the QoL specifically applicable to MU based on a standardized score.

Quality Assessment of the Included Studies

The methodological quality of the 50 included primary studies was appraised using the JBI Critical Appraisal Checklist for Case Series. Overall, 26 studies (52%) were judged to be at low risk of bias, 22 (44%) at moderate risk, and two (4%) at high risk. Confidence in case identification was high across the body of evidence: the condition was diagnosed by standard, reliable methods (Q2) and through valid histological confirmation (Q3) in almost all studies. The principal limitations were inherent to the retrospective case-series design-consecutive enrolment (Q4) was frequently unclear, and the completeness of outcome ascertainment and follow-up (Q8) was inconsistently reported, particularly in smaller or older series. Appropriate statistical analysis (Q10) was also often lacking, reflecting the predominantly descriptive nature of the included reports. These limitations were considered when interpreting the pooled estimates, which should be regarded as low- to very-low-certainty evidence (Table 2).

**Table 2:** Risk of bias (RoB) table

#	Study	Q1 Inclusion criteria	Q2 Standard condition measurement	Q3 Valid identification	Q4 Consecutive inclusion	Q5 Complete inclusion	Q6 Demographics reported	Q7 Clinical info reported	Q8 Outcomes/follow-up	Q9 Site/clinic info	Q10 Statistical analysis	Yes (/10)	Overall RoB
1	Al-Zacko 2013 [27]	Y	Y	Y	Y	Y	Y	Y	Y	Y	U	9	Low
2	Arons 1965 [10]	U	U	U	N	N	Y	Y	U	Y	U	3	High
3	Aydoğdu 2005 [28]	Y	Y	Y	U	Y	Y	Y	Y	Y	U	8	Low
4	Kumar 2024 [49]	Y	Y	Y	Y	Y	Y	Y	Y	Y	Y	9	Low
5	Baldursson 1999 [29]	U	Y	Y	Y	Y	Y	Y	Y	Y	Y	9	Low
6	Bang 2018 [6]	U	U	Y	U	U	U	Y	U	Y	U	3	High
7	Barr 1983 [12]	Y	Y	Y	U	U	Y	Y	Y	Y	N	7	Moderate
8	Bozkurt 2010 [11]	Y	Y	Y	U	Y	Y	Y	U	Y	U	7	Moderate
9	Burusapat 2021 [23]	Y	Y	Y	U	Y	Y	Y	Y	Y	Y	9	Low
10	Challa 2014 [13]	Y	Y	Y	U	U	Y	Y	Y	Y	U	7	Moderate
11	Chalya 2012 [30]	Y	Y	Y	U	U	Y	Y	U	Y	Y	7	Moderate
12	Chaturvedi 2019 [31]	Y	Y	Y	Y	Y	Y	Y	Y	Y	Y	10	Low
13	Chen 2020 [32]	U	Y	Y	U	Y	Y	Y	Y	Y	U	7	Moderate
14	Combemale 2007 [33]	U	Y	Y	Y	Y	Y	Y	U	Y	U	7	Moderate
15	Copcu 2003 [34]	Y	Y	Y	Y	Y	Y	Y	Y	Y	U	9	Low
16	Das 2015 [35]	Y	Y	Y	Y	Y	Y	Y	Y	Y	U	9	Low
17	Edwards 1989 [36]	Y	Y	Y	U	Y	Y	Y	Y	Y	Y	9	Low
18	Ehsani 2016 [9]	Y	Y	Y	U	U	Y	Y	U	Y	U	6	Moderate
19	Eroğlu 1997 [37]	U	Y	Y	N	N	Y	Y	Y	Y	Y	8	Moderate
20	Fahim 2022 [38]	U	U	U	U	U	Y	Y	N	Y	Y	5	Moderate
21	Ghalambor 2007 [39]	Y	Y	Y	Y	U	Y	Y	Y	Y	U	8	Low
22	Gül 2006 [7]	Y	Y	U	U	Y	Y	Y	U	Y	Y	7	Moderate
23	Guo 2018 [40]	Y	Y	Y	U	Y	Y	Y	Y	Y	Y	9	Low
24	Gupta 2023 [41]	Y	Y	Y	N	N	Y	Y	N	Y	Y	7	Moderate
25	Hahn 1990 [42]	Y	Y	Y	U	U	Y	Y	U	Y	U	6	Moderate
26	Huang 2010 [43]	Y	Y	Y	U	Y	Y	Y	U	Y	N	7	Moderate
27	Jiburum 2016 [44]	Y	Y	Y	U	Y	Y	Y	U	Y	U	7	Moderate
28	Kadir 2007 [45]	Y	Y	Y	Y	Y	Y	Y	U	Y	U	8	Low
29	Kasse 1999 [47]	Y	Y	U	U	Y	Y	Y	U	Y	U	6	Moderate
30	Kerr-Valentic 2009 [48]	Y	Y	Y	N	N	Y	Y	Y	Y	Y	8	Low
31	Lifeso 1990 [50]	Y	Y	Y	U	Y	Y	Y	Y	Y	Y	10	Low
32	Liu 2016 [51]	Y	Y	Y	U	Y	Y	Y	U	Y	Y	8	Low
33	Luo 2025 [52]	Y	Y	Y	U	U	Y	Y	Y	Y	Y	8	Low
34	Metwally 2017 [53]	Y	Y	Y	U	Y	Y	Y	Y	Y	Y	9	Low
35	Mousa 2022 [54]	Y	Y	Y	U	Y	Y	Y	Y	Y	U	8	Low
36	Onah 2006 [55]	Y	Y	Y	U	N	Y	Y	N	Y	N	6	Moderate
37	Oruç 2017 [56]	Y	Y	Y	Y	Y	Y	Y	Y	Y	U	9	Low
38	Ozek 2001 [57]	Y	Y	Y	N	N	Y	Y	Y	Y	N	7	Moderate
39	Ozinko 2017 [58]	Y	Y	Y	Y	Y	Y	Y	N	Y	N	8	Low
40	Sadegh Fazeli 2013 [59]	Y	Y	Y	U	Y	Y	Y	U	Y	Y	8	Low
41	Shahla 2009 [60]	Y	Y	Y	U	U	Y	Y	Y	Y	N	7	Moderate
42	Shen 2015 [61]	Y	Y	Y	U	Y	Y	Y	Y	Y	U	8	Low
43	Smith 2001 [62]	Y	Y	Y	U	U	Y	Y	U	Y	U	6	Moderate
44	Stromberg 1977 [63]	U	Y	Y	U	U	Y	Y	Y	Y	Y	7	Moderate
45	Tiftikcioglu 2010 [64]	Y	Y	Y	U	U	Y	Y	Y	Y	Y	8	Low
46	Tahir 2012 [15]	Y	Y	Y	U	U	Y	Y	Y	Y	Y	8	Low
47	Xiang 2019 [65]	U	Y	Y	U	Y	Y	Y	Y	Y	Y	8	Low
48	Xiao 2019 [66]	Y	Y	U	U	U	Y	Y	Y	Y	Y	7	Moderate
49	Karasoy Yesilada 2013 [46]	Y	Y	Y	U	Y	Y	Y	U	Y	U	7	Moderate
50	Yoo 2014 [67]	Y	Y	Y	U	Y	Y	Y	Y	Y	Y	9	Low
51	Yu 2013 [68]	Y	Y	Y	U	Y	Y	Y	Y	Y	Y	9	Low

**Figure 9:**
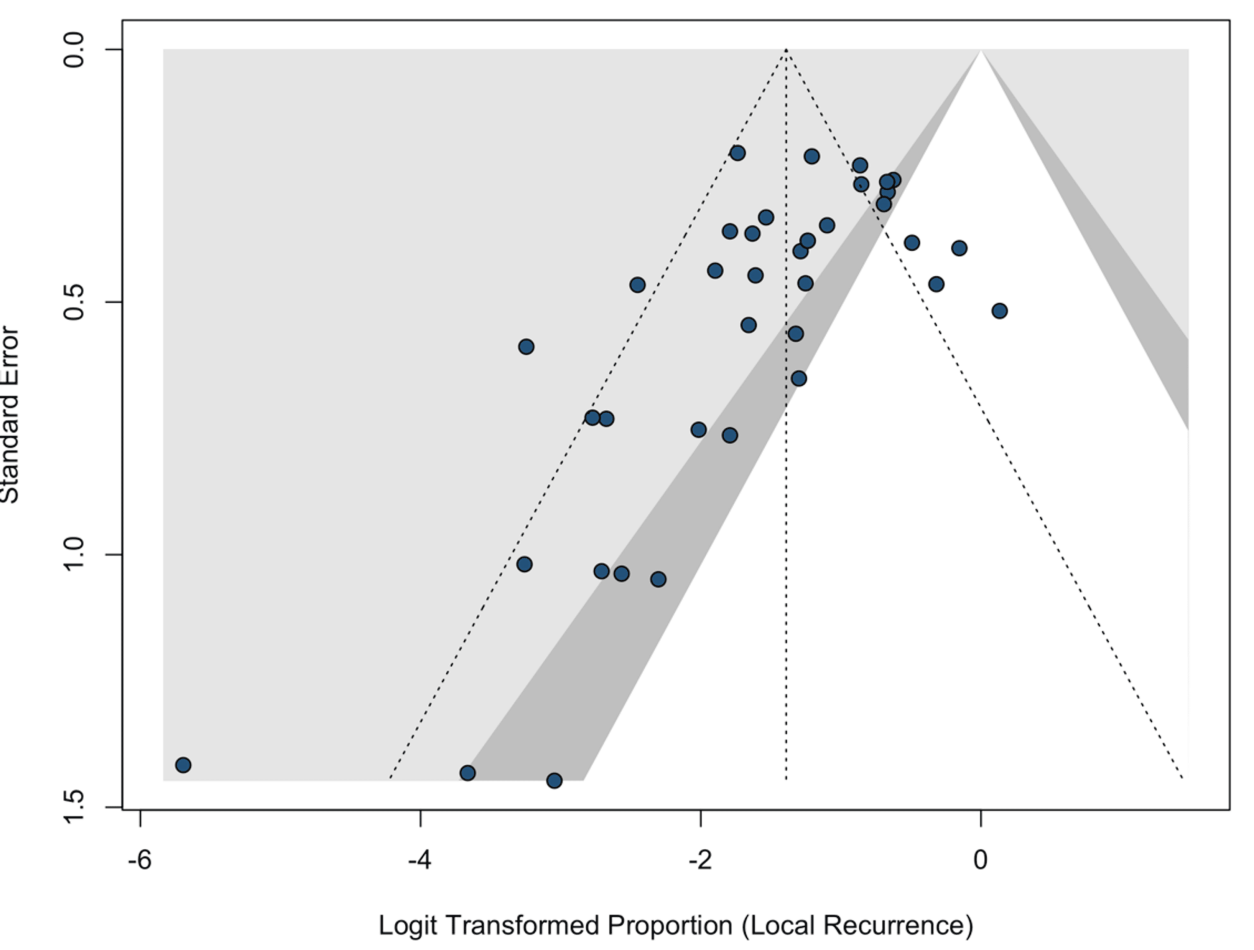
Funnel plot of local recurrence References [6,11-13,15,23,27-31,33-37,39,40,42,43,45-57,60,64,65,67,68]

The funnel plot of local recurrence is shown in Figure 9.

Discussion

This systematic review and meta-analysis summarizes the pooled clinicopathological features and oncological outcomes in MU. This is based on available studies, which are mostly retrospective. As this condition is mostly reported from low- and middle-income countries of the tropics or subtropics, this condition is under-represented in available cancer registries. In this systematic review and meta-analysis of 51 studies comprising approximately 2,388 patients, SCC (95.7%) arose chiefly in the lower limb after a characteristically long latency (median 29.5 years). Nodal metastasis at diagnosis was reported in 37 studies (1,656 patients), with a pooled prevalence of 19% (95% CI 15%-24%). Local recurrence was assessed across 38 studies (1,787 patients) and showed a comparable pooled proportion of 20% (95% CI 16%-24%), with moderate-to-substantial heterogeneity. Pooled five-year overall survival was 61% (95% CI 49%-72%). Local recurrence was the most robust estimate; however, substantial heterogeneity and wide prediction intervals reflect inconsistent definitions and ascertainment across the included series.

Earlier studies have shown that, among clinicopathological factors, higher histological grade was associated with recurrence and mortality, and regional lymph node involvement at diagnosis was a strong and reproducible predictor of disease-specific death; in contrast, the prognostic influence of tumor size was inconsistent, and perineural invasion-though significant in the single dedicated study-remained inadequately replicated. The present meta-analysis shows that adding adjuvant RT to surgery did not demonstrate a significant effect on local recurrence, and the available comparison was severely confounded by indication of use of RT. Overall, the certainty of evidence was low to very low, reflecting the retrospective, observational nature of the source data.

The definition of MU has broadened over time to encompass any malignant transformation occurring in a chronic wound, scar, or chronically inflamed skin, regardless of the initial cause of injury, with SCC being the predominant histological type. The data from the German Marjolin registry had a preponderance of chronic venous ulcers [70]. This wider usage is consistent with the recent literature, which characterizes it as an aggressive cutaneous malignancy arising in chronic, non-healing wounds. The broader definition is biologically plausible because malignant change is driven by the chronic-wound microenvironment: chronic inflammation and a high reactive-oxygen-species burden, repeated cycles of injury that promote DNA damage, and impaired immune surveillance within avascular tissue that limits immune-cell access, allowing transformed cells to evade detection [71]. Considering the aggressive biology and clinical presentation, clustering SCC with other histological subtypes may not be useful in terms of treatment and prognostic implications. It is also observed that the clinical behavior can be different in different etiological causes like burn, radiation, and venous stasis ulcer. Latency also forms part of the working definition. The most widely used convention requires a minimum latency of approximately one year from the inciting injury [27,40]. Lesions arising within a year-or, in some definitions, within one to two years-are termed "acute Marjolin's ulcer" by a few authors, but this is not widely accepted [9,13,40]. The one-year threshold is itself a pragmatic choice: it carries no biological basis and is applied inconsistently across studies [13]. The latency of the SCC subtype has been reported to average around a decade or longer [1,15,52]. Some, on the contrary, have reported a very large latent period running to 70 years and above [28,36]. A shorter latency has been described for non-SCC lesions: in one series, the mean latency of SCC was 13.42 years (range, six months-54 years), melanoma was 2.47 years (range, three months-10 years), and a single BCC arose after three years [61]. Shorter latency periods have been reported from certain geographic regions like sub-Saharan Africa [72]. Such heterogeneity in case definition and inclusion in the reported studies for this rare condition is a challenge for evidence synthesis.

Despite the rarity of the condition, a geographical predilection in its occurrence is evident in the reported studies. In certain countries, like Nigeria, it constitutes 30% of skin cancer cases [8,44,55,58,73,74]. The most common histology is SCC, which has a higher chance of lymph nodal metastasis and is more aggressive, with a worse survival rate [41]. SCCs developing on burn scars were reported to be more common in the lower extremities. A change in the character of a long-standing wound is the key clinical clue to malignant transformation. This may take the form of recent enlargement, new ulceration, or an exophytic or nodular growth, often with raised, indurated, or everted margins and a friable, slough- or necrosis-covered base. Bleeding on minimal contact, foul-smelling discharge, and excessive granulation tissue are common, and the emergence of pain in a previously painless scar is an early warning sign warranting suspicion [75]. A significant difference between MU- and non-MU-SCC was found regarding metastases (p = 0.001) [7]. Although classically a surgical margin of 2 cm has been widely used for the resection of primary MU, an increased margin of 2.5 cm has been advocated for resection in recurrent cases [39]. Compared to MU in post-radiation causes, post-burn MU had a higher incidence of nodes and more local recurrence, whereas post-RT had more regional recurrence in lymph nodes [36]. Regional lymph node involvement is relatively frequent at presentation, reflecting the aggressive biological behavior of the tumor. Reported rates of nodal metastasis exceed those of conventional cutaneous SCC (cSCC), typically ranging from 20% to 30%. Advanced cases may also present with distant metastases, most commonly to the lungs, liver, and bone. Local invasion of deeper structures such as fascia, muscle, or bone is not uncommon, particularly in neglected lesions [19,35,75]. Adverse pathological features such as large tumor size (>10 cm), high-grade lesion, location, previous history of recurrence, lymph node metastasis, joint involvement, distant metastasis, and lymphovascular or perineural invasion are associated with higher rates of recurrence and mortality [23,30,31,40,41,76]. These factors, combined with delayed diagnosis and the absence of multidisciplinary care, contribute to the overall poorer prognosis observed in patients with MU.

Surgery remains the cornerstone of management for MU. WLE is the preferred treatment for localized disease, with most authors recommending excision > 2 margins of clinically normal tissue and resection extending to the deep fascia or the next uninvolved anatomical plane to achieve adequate oncological clearance [11,27,31]. Amputation is generally reserved for advanced lesions with extensive involvement of bone, joints, or major neurovascular structures, as well as tumors in which adequate oncological clearance cannot be achieved with limb-sparing surgery [49]. Following tumor resection, reconstruction is commonly achieved using split-thickness skin grafts for superficial defects, whereas local, regional, or free flaps may be required for larger defects and for coverage of exposed vital structures. Management of regional lymph nodes remains controversial. Most authors advocate therapeutic lymph node dissection only in patients with clinically, radiologically, or cytologically proven nodal metastasis and do not recommend routine elective or prophylactic lymph node dissection in clinically node-negative patients because of the lack of demonstrated survival benefit [31,41]. Lymph node dissection is recommended for clinically palpable lymphadenopathy [77]. Sentinel lymph node biopsy has been proposed as a staging modality in selected high-risk patients without clinically evident nodal disease; however, evidence supporting its routine use remains limited, and its role remains undefined [6,78,79]. Recurrent disease should be managed aggressively, with wider margin or with repeat wide excision being appropriate for localized recurrences, whereas amputation may be necessary for recurrent lesions with extensive deep tissue or bony involvement. Time to recurrence varied from one month to 11 years (mean 21.7 months) [65].

The role of RT in the present review was heterogeneous. Various studies have used RT as a treatment modality either in definitive, adjuvant, or palliative settings [31,35,42,50,54,67]. The indications for RT included large inoperable disease (>10 cm), regional lymph node metastasis, poorly differentiated histology, positive lymph nodes after nodal dissection, and head-and-neck lesions with positive lymph nodes after regional lymph node dissection. In our meta-analysis reporting local recurrence by treatment arm, the addition of adjuvant RT to surgery was not associated with a significant change in local recurrence (log risk ratio 0.78 (-1.52-3.12)). This estimate should not be interpreted as a treatment effect: the comparison was severely confounded by indication, as RT was selectively administered to patients with higher-risk, node-positive, or locally advanced disease, and several arms contained zero events, leading to wide and unstable CIs. The evidence is therefore insufficient to establish whether adjuvant RT alters local control, and a true estimate of benefit would require individual-patient data adjusted for stage, grade, margin status, and nodal involvement, or ideally prospective evaluation. Consequently, RT is best regarded as a selectively applied adjunct for high-risk or unresectable disease rather than a routine component of management. Several authors have suggested prophylactic lymph node dissection based on histological tumor grade or the suitability of sentinel lymph node dissection. Prophylactic nodal treatment seems rational because of the aggressive biological behavior of MU; it has been recommended that regional lymph node dissection should be performed only in clinically positive nodal basins or if lymph nodes are positive on fine-needle aspiration histology [6].

Despite the aggressive nature of MU and the substantial morbidity associated with its treatment, the impact of surgical management on patient QoL remains largely unexplored [80]. In contrast, a limited body of literature exists for cSCC and non-melanoma skin cancers. Chren et al. demonstrated significant improvements in symptom-related, emotional, and functional QoL domains following surgical excision and Mohs surgery for non-melanoma skin cancers, highlighting the importance of patient-reported outcomes alongside traditional oncological endpoints [81]. Similarly, Burdon-Jones and Gibbons developed and validated the Skin Cancer Quality of Life Impact Tool (SCQOLIT), identifying key patient concerns including fear of recurrence, anxiety, need for reassurance, treatment-related disfigurement, and psychosocial distress. However, even within cSCC, QoL research remains limited. In a structured review, Starkings et al. identified only seven studies evaluating QoL in patients with advanced or high-risk cSCC and concluded that the available evidence is sparse, heterogeneous, and insufficient to fully characterize patient experience [82,83]. MU represents a particularly aggressive subset of cSCC, frequently arising in chronic scars or wounds and often requiring extensive excision, complex reconstruction, or even amputation. These disease-specific characteristics are likely to exert a profound impact on physical function, body image, emotional well-being, and social reintegration. Nevertheless, dedicated studies evaluating QoL outcomes following surgical treatment of MU are virtually absent from the published literature. This significant knowledge gap underscores the need for prospective assessment of patient-reported QoL outcomes in this unique and clinically challenging population.

This review has several strengths, including a comprehensive search, formal risk of bias appraisal, and the largest quantitative synthesis of oncological outcomes and prognostic factors in MU to date. Nevertheless, there are important limitations. The evidence base consists almost entirely of retrospective, single-arm case series, so the overall certainty of evidence was low to very low, and the pooled estimates are susceptible to selection, detection, and reporting biases. Substantial statistical heterogeneity was present for regional nodal (I² = 80%) and distant (I² = 87%) metastasis, driven largely by inconsistent outcome definitions and by variable ascertainment-nodal metastasis was variably recorded at diagnosis, on elective or sentinel-node dissection, or during follow-up-such that the corresponding pooled proportions are best read alongside their wide prediction intervals rather than as single point estimates. Finally, because MU is reported predominantly from low- and middle-income tropical and subtropical regions and is under-represented in cancer registries, the literature is geographically uneven, and the exclusion of non-English studies and series of fewer than 10 patients may have introduced further selection bias.

## Conclusions

MU is an aggressive, predominantly squamous cell malignancy of the lower limb arising after a prolonged latency, with a pooled local recurrence rate of approximately 19%, regional nodal and distant metastasis rates of approximately 16% and 8%, and a five-year overall survival of roughly 57%. Adjuvant RT showed no demonstrable effect on local control in confounded data. These findings, derived from low- to very-low-certainty evidence, support WLE with clear margins as the cornerstone of treatment and therapeutic rather than routine prophylactic nodal management, while underscoring that the prognostic and therapeutic questions central to this disease remain inadequately answered. In the absence of high-quality, prospective studies, the present systematic review and meta-analysis fill a gap in the literature by transparently pooling and correlating clinicopathological factors with patient-, tumor-, and treatment-related factors, as well as their outcomes. Given the prediction interval and long latency, the significant impact of morbidity on QoL in patients with MU needs to be addressed in the future through objective assessment of QoL across various treatment modalities.

## Appendices

Search Strategy

A comprehensive search-designed in accordance with PRISMA-P 2015 Item 10 was conducted from each database's earliest inception to the search date by an experienced medical librarian. The following databases will be searched:

Database

Search Strategy/Terms

PubMed

("Marjolin ulcer"[tiab] OR "Marjolin's ulcer"[tiab] OR Marjolin[tiab] OR "scar carcinoma"[tiab] OR ("malignant transformation"[tiab] AND scar[tiab]) OR ("burn scar"[tiab] AND "squamous cell carcinoma"[tiab]) OR (post-burn[tiab] AND carcinoma[tiab]) OR (postburn[tiab] AND carcinoma[tiab]) OR ("chronic wound"[tiab] AND carcinoma[tiab]) OR (osteomyelitis[tiab] AND (sinus[tiab] OR fistula[tiab]) AND carcinoma[tiab]) OR ("pressure ulcer"[tiab] AND "squamous cell carcinoma"[tiab]) OR ("Carcinoma, Squamous Cell"[Mesh] AND "Cicatrix"[Mesh]) OR ("Skin Ulcer"[Mesh] AND "Carcinoma, Squamous Cell"[Mesh])) AND ("squamous cell carcinoma"[tiab] OR SCC[tiab] OR "Carcinoma, Squamous Cell"[Mesh] OR "spindle cell carcinoma"[tiab] OR "verrucous carcinoma"[tiab] OR "skin cancer"[tiab]) AND ("perineural invasion"[tiab] OR PNI[tiab] OR Breslow[tiab] OR "tumour depth"[tiab] OR "tumor depth"[tiab] OR "depth of invasion"[tiab] OR "poor differentiation"[tiab] OR "poorly differentiated"[tiab] OR "tumour diameter"[tiab] OR "tumor diameter"[tiab] OR "tumour size"[tiab] OR "tumor size"[tiab] OR "lymphovascular invasion"[tiab] OR LVI[tiab] OR immunosuppression[tiab] OR immunocompromised[tiab] OR "organ transplant"[tiab] OR location[tiab] OR "latency period"[tiab] OR "scar duration"[tiab] OR ("malignant transformation"[tiab] AND latency[tiab]) OR "surgical margin"[tiab] OR "resection margin"[tiab] OR "margin status"[tiab]) AND (recurrence[tiab] OR recurrent[tiab] OR "local recurrence"[tiab] OR metastasis[tiab] OR metastases[tiab] OR metastatic[tiab] OR "nodal metastasis"[tiab] OR "lymph node metastasis"[tiab] OR "regional metastasis"[tiab] OR survival[tiab] OR "overall survival"[tiab] OR "disease-specific survival"[tiab] OR "disease-specific death"[tiab] OR "cause-specific survival"[tiab] OR "disease-free survival"[tiab] OR "recurrence-free survival"[tiab] OR prognos*[tiab] OR mortality[tiab] OR death[tiab] OR fatal*[tiab] OR "Treatment Outcome"[Mesh] OR (Marjolin[tiab] AND (tumor[tiab] OR tumour[tiab] OR carcinoma[tiab])))

EMBASE (Ovid)

( 'marjolin ulcer':ti,ab,kw OR 'marjolin''s ulcer':ti,ab,kw OR marjolin:ti,ab,kw OR 'scar carcinoma':ti,ab,kw ) AND ( 'squamous cell carcinoma'/exp OR 'squamous cell carcinoma':ti,ab,kw OR SCC:ti,ab,kw )

Cochrane Central Register of Controlled Trials (CENTRAL)

1 Trial matching ( "Marjolin ulcer":ti,ab,kw OR "Marjolin's ulcer":ti,ab,kw OR Marjolin:ti,ab,kw OR "scar carcinoma":ti,ab,kw ) AND ( "squamous cell carcinoma":ti,ab,kw OR SCC:ti,ab,kw OR carcinoma:ti,ab,kw ) in Title Abstract Keyword

Scopus

( TITLE-ABS-KEY("Marjolin ulcer") OR TITLE-ABS-KEY("Marjolin's ulcer") OR TITLE-ABS-KEY(Marjolin) OR TITLE-ABS-KEY("scar carcinoma") OR (TITLE-ABS-KEY("malignant transformation") AND TITLE-ABS-KEY(scar)) OR (TITLE-ABS-KEY("burn scar") AND TITLE-ABS-KEY("squamous cell carcinoma")) OR (TITLE-ABS-KEY(post-burn) AND TITLE-ABS-KEY(carcinoma)) OR (TITLE-ABS-KEY(postburn) AND TITLE-ABS-KEY(carcinoma)) OR (TITLE-ABS-KEY("chronic wound") AND TITLE-ABS-KEY(carcinoma)) OR (TITLE-ABS-KEY(osteomyelitis) AND (TITLE-ABS-KEY(sinus) OR TITLE-ABS-KEY(fistula)) AND TITLE-ABS-KEY(carcinoma)) OR (TITLE-ABS-KEY("pressure ulcer") AND TITLE-ABS-KEY("squamous cell carcinoma")) ) AND ( TITLE-ABS-KEY("squamous cell carcinoma") OR TITLE-ABS-KEY(SCC) OR TITLE-ABS-KEY("spindle cell carcinoma") OR TITLE-ABS-KEY("verrucous carcinoma") OR TITLE-ABS-KEY("skin cancer") ) AND ( TITLE-ABS-KEY("perineural invasion") OR TITLE-ABS-KEY(PNI) OR TITLE-ABS-KEY(Breslow) OR TITLE-ABS-KEY("tumour depth") OR TITLE-ABS-KEY("tumor depth") OR TITLE-ABS-KEY("depth of invasion") OR TITLE-ABS-KEY("poor differentiation") OR TITLE-ABS-KEY("poorly differentiated") OR TITLE-ABS-KEY("tumour diameter") OR TITLE-ABS-KEY("tumor diameter") OR TITLE-ABS-KEY("tumour size") OR TITLE-ABS-KEY("tumor size") OR TITLE-ABS-KEY("lymphovascular invasion") OR TITLE-ABS-KEY(LVI) OR TITLE-ABS-KEY(immunosuppression) OR TITLE-ABS-KEY(immunocompromised) OR TITLE-ABS-KEY("organ transplant") OR TITLE-ABS-KEY(location) OR TITLE-ABS-KEY("latency period") OR TITLE-ABS-KEY("scar duration") OR (TITLE-ABS-KEY("malignant transformation") AND TITLE-ABS-KEY(latency)) OR TITLE-ABS-KEY("surgical margin") OR TITLE-ABS-KEY("resection margin") OR TITLE-ABS-KEY("margin status") ) AND ( TITLE-ABS-KEY(recurrence) OR TITLE-ABS-KEY(recurrent) OR TITLE-ABS-KEY("local recurrence") OR TITLE-ABS-KEY(metastasis) OR TITLE-ABS-KEY(metastases) OR TITLE-ABS-KEY(metastatic) OR TITLE-ABS-KEY("nodal metastasis") OR TITLE-ABS-KEY("lymph node metastasis") OR TITLE-ABS-KEY("regional metastasis") OR TITLE-ABS-KEY(survival) OR TITLE-ABS-KEY("overall survival") OR TITLE-ABS-KEY("disease-specific survival") OR TITLE-ABS-KEY("disease-specific death") OR TITLE-ABS-KEY("cause-specific survival") OR TITLE-ABS-KEY("disease-free survival") OR TITLE-ABS-KEY("recurrence-free survival") OR TITLE-ABS-KEY(prognos*) OR TITLE-ABS-KEY(mortality) OR TITLE-ABS-KEY(death) OR TITLE-ABS-KEY(fatal*) OR (TITLE-ABS-KEY(Marjolin) AND (TITLE-ABS-KEY(tumor) OR TITLE-ABS-KEY(tumour) OR TITLE-ABS-KEY(carcinoma))) )

Science Direct

Title, abstract, keywords: ("Marjolin ulcer" OR Marjolin) AND ("squamous cell carcinoma" OR SCC)

Google Scholar

Marjolin ulcer; malignant ulcer; squamous cell carcinoma; burn scar

Hand-searching

Reference lists of all included studies and relevant review articles; grey literature (WHO IRIS, OpenGrey, ProQuest Dissertations)

Search Terms

Core search terms will be built around three concept clusters combined with Boolean AND:

•        Cluster 1 - Condition: Marjolin* | "scar carcinoma" | "malignant ulcer" | "burn scar squamous" | "chronic wound malignancy" | "post-burn carcinoma" | "osteomyelitis sinus carcinoma" | "pressure ulcer carcinoma"

•        Cluster 2 - Histology: "squamous cell carcinoma" | SCC | "spindle cell carcinoma" | carcinoma

•        Cluster 3 - Outcomes: recur* | metastas* | survival | mortality | prognos* | "disease-specific death" | "disease-free survival" | "overall survival"

MeSH/EMTREE controlled vocabulary terms will supplement free-text searching. The complete, date-stamped, peer-reviewed search strategy (following PRESS checklist) will be appended to the final manuscript.

No Language Restriction

No language restrictions will be imposed. Non-English publications will be translated using validated machine translation tools supplemented by human verification where feasible.
